# Comparative Chloroplast Genomics of Seven Endangered *Cypripedium* Species and Phylogenetic Relationships of Orchidaceae

**DOI:** 10.3389/fpls.2022.911702

**Published:** 2022-06-22

**Authors:** Jun-Yi Zhang, Min Liao, Yue-Hong Cheng, Yu Feng, Wen-Bing Ju, Heng-Ning Deng, Xiong Li, Andelka Plenković-Moraj, Bo Xu

**Affiliations:** ^1^China-Croatia “Belt and Road” Joint Laboratory on Biodiversity and Ecosystem Services, CAS Key Laboratory of Mountain Ecological Restoration and Bioresource Utilization & Ecological Restoration and Biodiversity Conservation Key Laboratory of Sichuan Province, Chengdu Institute of Biology, Chinese Academy of Sciences, Chengdu, China; ^2^College of Life Sciences, Chongqing Normal University, Chongqing, China; ^3^College of Life Sciences, University of Chinese Academy of Sciences, Beijing, China; ^4^Wolong National Natural Reserve Administration Bureau, Sichuan, China; ^5^Faculty of Science, University of Zagreb, Zagreb, Croatia

**Keywords:** *Cypripedium*, chloroplast genome, IR expansion, molecular markers, Orchidaceae, phylogenomics

## Abstract

The species in the genus *Cypripedium* (Orchidaceae) are considered endangered, mainly distributed in the temperate regions of the Northern Hemisphere, with high ornamental and economic value. Despite previous extensive studies based on both morphology and molecular data, species and sections relationships within *Cypripedium* remain controversial. Here, we employed two newly generated *Cypripedium* chloroplast genomes with five other published genomes to elucidate their genomic characteristics. The two genomes were 162,773–207,142 bp in length and contained 128–130 genes, including 82–84 protein-coding genes, 38 tRNA genes, and 8 rRNA genes. We identified 2,192 simple sequence repeats, 786 large repeat sequences, and 7,929 variable loci. The increase of repeat sequences (simple sequence repeats and large repeat sequences) causes a significant amplification in the chloroplast genome size of *Cypripedium*. The expansion of the IR region led to the pseudogenization or loss of genes in the SSC region. In addition, we identified 12 highly polymorphic loci (Pi > 0.09) suitable for inferring the phylogeny of *Cypripedium* species. Based on data sets of whole chloroplast genomes (IRa excluded) and protein-coding sequences, a well-supported phylogenetic tree was reconstructed, strongly supporting the five subfamilies of Orchidaceae and the genus *Cypripedium* as monophyletic taxa. Our findings also supported that *C. palangshanense* belonged to sect. *Palangshanensia* rather than sect. *Retinervia*. This study also enriched the genomic resources of *Cypripedium*, which may help to promote the conservation efforts of these endangered species.

## Introduction

Orchidaceae is one of the two largest families of angiosperms, consisting of approximately 880 genera and 27,000 species, accounting for 8% of all vascular plant species and growing in a wide range of habitats worldwide (Chase et al., 2003, 2015). The genus *Cypripedium* L. consisted of approximately 51 species with unique colorful labellum (known as lady’s slipper), which mainly distributed in subtropical to temperate in the Northern Hemisphere (Cribb, 1997; Chen and Cribb, 2005; Chen et al., 2013). More than two-thirds of these species were native to China, especially in Yunnan, Sichuan and Tibet (Chen et al., 2013). In the wild, many species have become rare and endangered due to shrinking natural habitats and over-collection for gardens and herbarium (Nelson, 1994; Farrell, 1999; Szlachetko et al., 2020). Thus, this genus is currently listed on the Convention on International Trade in Endangered Species of Wild Fauna and Flora, CITES Appendix II (Mcgough et al., 2006).

A robust and well-resolved orchid family phylogeny is fundamental to understanding the evolution and diversification of Orchidaceae, including individual traits, species diversification and conservation. Over the past two decades, growing evidence of molecular markers (such as plastid markers, nuclear ribosomal DNA, mitochondrial or low-copy nuclear genes) has greatly advanced our understanding of orchid relationships, clarifying relationships among orchid subfamilies with morphologically confused taxa (Górniak et al., 2010; Guo et al., 2012; Chase et al., 2015; Deng et al., 2015; Givnish et al., 2015; Li et al., 2016). However, the relationship between subfamily Cypripedioideae and Vanilloideae has been controversial. For example, Deng et al. (2015); Givnish et al. (2015); Li et al. (2016) indicated that subfamily Vanilloideae (vs. Cypripedioideae; Cameron et al., 1999; Li et al., 2019) belonged to the second diverged taxa of the orchid family. In addition, recent molecular phylogenetic studies within the genus *Cypripedium* (nuclear ribosomal ITS, low copy nuclear gene (*ACO*) and chloroplast genes (*matK*, *rbcL*, *trnH*-*psbA*, *atpI*-*atpH*, *trnS*-*trnfM* and *trnL*-*F*) supported that the genus was monophyletic and roughly divided into 15 sections (Li et al., 2011; Chen et al., 2013; Szlachetko et al., 2020). But the internal structure is not well resolved, such as the sect. *Retinervia* and sect. *Palangshanensia* (Cox et al., 1997; Cribb, 1997; Eccarius, 2009).

Chloroplast is an important organelle that promotes the growth and development of most plants and plays an important role in the biosynthesis of plant carbohydrates, proteins and lipids (Daniell et al., 2016). The chloroplast genome consists of a helical, double-stranded genome with the aptitude to replicate independently of the nuclear genome (Palmer, 1985; Abdullah et al., 2021). Chloroplast genomes of land plants are typically circular DNA molecules with highly conserved regions, gene content, and gene order (Wicke et al., 2011). The average chloroplast genome size of land plants is 151 kb, with most species ranging from 130–170 kb in length, and the average GC content is 36.3% (Guo et al., 2021). A typical chloroplast genome consists of a pair of inverted repeats (IR) regions separated by a large single copy (LSC) region and a small single copy (SSC) region (Sugiura, 1992). In recent years, with the rapid development of next-generation sequencing technology, thousands of complete chloroplast genomes from various land plants have been sequenced (Yu et al., 2017), among which 394 were from Orchidaceae (NCBI^1^, accessed on April 7, 2021). To date, only five chloroplast genomes have been reported for this genus, *Cypripedium japonicum* Thunberg (Kim et al., 2015), *C. formosanum* Hayata (Lin et al., 2015), *C. calceolus* L. (Zhang et al., 2019), *C. subtropicum* Chen & Lang (Guo et al., 2021) and *C. tibeticum* King ex Rolfe (Guo et al., 2021). The lengths of all reported chloroplast genomes of *Cypripedium* (174,417–212,668 bp) were higher than the average of land plants (151 kb). The chloroplast genome of *C. subtropicum* was 212,668 bp, which is the largest known genome of orchids and the sixth largest of terrestrial plants (Guo et al., 2021). Chloroplast genome sequences have been widely recognized for phylogenetic and divergence history studies in flowering plants (Tonti-Filippini et al., 2017; Feng et al., 2020). Also, chloroplast genomes can provide unique and substantial information for the analysis of plant systematics and evolutionary relationships with matrilineal inheritance characteristics (Wang et al., 2016). Meanwhile, highly variable loci identified in the chloroplast genome can make significant contributions to future phylogenetic studies of the genus (Chen et al., 2015).

In the present study, we generated two newly sequenced chloroplast genomes of *Cypripedium* species and performed comparative genomic analysis in combination with five other published chloroplast genomes from this genus. We also included 47 whole plastid genomes and plastid protein-coding genes (CDSs) representing five subfamilies of Orchidaceae for phylogenetic analysis. Our aims were to (1) explore the patterns of long sequence repeats (LSRs) and simple sequence repeats (SSRs) in seven *Cypripedium* plastid genomes that cause significant expansion and contraction of the genome; (2) identify polymorphic loci for future phylogenetic inference of the genus *Cypripedium*; and (3) elucidate the molecular evolution and phylogenetic relationships of *Cypripedium* species and Orchidaceae.

## Materials and Methods

### Sample Materials Collection, DNA Extraction, and Sequencing

Fresh leaf samples of *Cypripedium palangshanense* Tang & Wang and *C. debile* Rchb. were collected from the native environment in Wolong National Nature Reserve, Sichuan, China. The collected leaf samples were kept in silica gel and stored at the Herbarium of Chengdu Institute of Biology (CDBI). Total genomic DNA was extracted using a modified cetyltrimethylammonium bromide (CTAB) method (Allen et al., 2006). Sheared low molecular weight DNA fragments were used to construct paired-end (PE 150) libraries according to the protocol of the Illumina manual (Illumina, CA, United States). Completed libraries were pooled and sequenced in the Illumina NovaSeq 6000 platform with 350 bp insert size (Berry Genomics, Beijing, China).

### Chloroplast Genome Assembly and Annotation

Approximately 19 Gb of clean data for *Cypripedium palangshanense* and 15 Gb of clean data for *C. debile* were used to assemble the chloroplast genomes with GetOrganelle v1.7.2 (Jin et al., 2020). Bandage (Wick et al., 2015) was used to identify the circular maps to assess the quality of the assembly. The average coverage for the assembled chloroplast genomes was 770.6 × and 510.1 × for *C. palangshanense* and *C. debile*, respectively. The assembled chloroplast genomes were annotated using PGA (Qu et al., 2019) with chloroplast genomes of *C. calceolus* (NC_045400) and *C. japonicum* (NC_027227) as reference sequences. To quantify IR boundaries, raw sequencing reads were remapped to the 600-bp surroundings of the IR ends. Manual correction of genes with missing start and stop codons in annotations was performed using Geneious Prime 2021 (Biomatters Ltd., Auckland, New Zealand). The circular chloroplast genome maps were visualized using OGDRAW v1.3.132 (Greiner et al., 2019).

### Comparative Analysis of *Cypripedium* Chloroplast Genomes

The sequence of *Cypripedium calceolus*, *C. japonicum*, *C. subtropicum*, *C. tibeticum* and *C. formosanum* was included in the comparative chloroplast genome analysis. The base content was determined using DNA Baser Sequence Assembler v5.15^2^. To identify highly variable regions, the seven chloroplast genomes were aligned using the MAFFT v7.475 (Katoh and Standley, 2013) with default parameters. The number of polymorphic sites and nucleotide variability (Pi) were evaluated using a sliding window with 200-bp step size and a 600-bp window length implemented in DnaSP v5.10.1 (Librado and Rozas, 2009). Full alignments with annotation were visualized using the mVISTA software (Frazer et al., 2004), and gene arrangement was further analyzed by the Mauve alignment plugin in Geneious Prime 2021 (Biomatters Ltd., Auckland, New Zealand). The junction of chloroplast genomes was analyzed in IRscope (Amiryousefi et al., 2018) to visualize the expansion and contraction of inverted repeats.

### Repeat Sequences Analysis

Large sequence repeats (LSRs), including forward (F), reverse (R), complement (C) and palindrome (P) sequence repeats, were identified in whole chloroplast genome, LSC, SSC and IR regions using REPuter (Kurtz et al., 2001; Hamming distance = 3 and minimum repeat size of 30 bp). Simple sequence repeats (SSRs) (≥ 10 bp) were detected using MISA (Beier et al., 2017) with the minimum thresholds for mononucleotide, dinucleotide, trinucleotide, tetranucleotide, pentanucleotide, and hexanucleotide were set to 10, 5, 4, 3, 3, and 3, respectively. In addition, tandem repeats were identified with Tandem Repeats Finder v4.09 (Benson, 1999) with default parameters. The overlapped repeats of the results were removed manually.

### Phylogenetic Analysis

The whole chloroplast genome sequences and protein-coding sequences of 45 Orchidaceae (Apostasioideae, Vanilloideae, Cypripedioideae, Orchidoideae, and Epidendroideae) species were downloaded from the National Center for Biotechnology Information Search database (Supplementary Table 1). Phylogenetic relationships within Orchidaceae were reconstructed in combination with the 45 published accessions and the two newly generated *Cypripedium* chloroplast genomes. Two species of family Iridaceae (*Iris dichotoma* Pall., MK593157) and Amaryllidaceae (*Lycoris sanguinea* Maxim., NC_047453) were included as outgroups in the phylogenetic analysis. Before constructing the phylogenetic tree, we manually corrected all inversions using Geneious Prime 2021 to obtain consistent gene and base order. Phylogenetic analyses were performed based on the following two data sets: (1) the complete chloroplast genome sequences (IRa excluded); (2) the extracted sequences representing all coding sequences (CDSs). We used MAFFT v7.475 (Katoh and Standley, 2013) with default parameters to obtain aligned whole chloroplast genomes (IRa excluded) and CDSs, as well as manual adjustments where necessary. Three different methods including Maximum parsimony (MP), Maximum likelihood (ML) and Bayesian inference (BI) were employed in the phylogenetic analysis. The MP analysis based on the concatenated data set was carried out using PAUP v4.0b10 (Swofford, 2003), with a heuristic search with 1,000 random taxon stepwise addition sequences, tree bisection reconnection branch swapping, and 1,000 bootstrap replications. The ML analysis was performed using IQ-TREE v.1.4.241 (Nguyen et al., 2014), the ModelFinder in IQ-TREE tested a total of 286 DNA models and chose TIM + F + R3 as the best-fit nucleotide substitution model for the two data matrices, and branch support was estimated using 2,000 replicates of both SH-like approximate likelihood-ratio test (SH-aLRT) (Guindon et al., 2010) and the ultrafast bootstrapping algorithm (UFboot) (Minh et al., 2013). For BI analysis, the best-fit nucleotide substitution models (GTR + I + G) for the two data matrices were chosen based on the corrected Akaike Information Criterion (AICc) using jModeltest v2.1.6 (Posada, 2008) software. The BI analysis was conducted using MrBayes v3.2.7a (Ronquist and Huelsenbeck, 2003) with two parallel runs (20 million generations). The first 25% of trees from all runs were discarded as burn-in. The results were visualized in Figtree v1.4.4^3^.

## Results

### Chloroplast Genomes of *Cypripedium palangshanense* and *Cypripedium debile*

**FIGURE 1:**
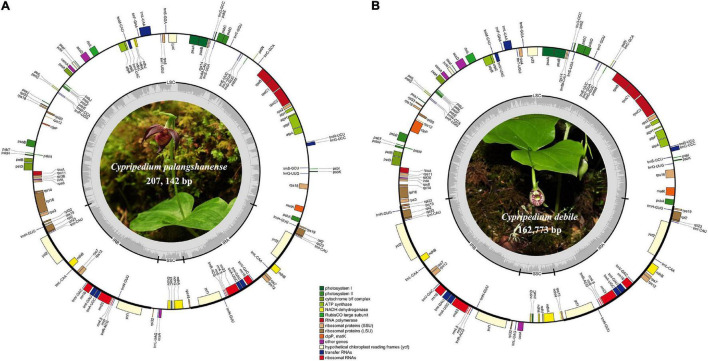
Chloroplast genome maps of *C. palangshanense* **(A)** and *C. debile* **(B)**. Genes of different functional groups are shown in colored bars. The inner circle (dashed gray area) indicates the proportional GC content of the corresponding genes. Regions of the large single-copy (LSC), small single-copy (SSC) and inverted repeats (IRA and IRB) are indicated.

We obtained the whole chloroplast genomes of 207,142 bp for *Cypripedium palangshanense* and 162,773 bp for *C. debile* (GenBank accession Nos. MW924110 and MW924111, respectively). The chloroplast genomes of these two species showed a typical quadripartite structure containing a pair of IRs separated by an SSC region and an LSC region (Figure 1). The IR boundaries were quantified by the remapping of short reads, which showed above 500 × for the IR ends and surrounding areas (Supplementary Datasheet). The LSC region of *C. palangshanense* expanded to 128,862 bp, similar to *C. subtropicum* (129,998 bp), while the LSC region of *C. debile* was 89,446 bp, the smallest of the published *Cypripedium* genomes (Table 1). The IR regions of the two species (34,415 and 31,639 bp, respectively) were slightly larger than the other five species, but the SSC regions (9,450 and 10,049 bp, respectively) were smaller than previously sequenced species (Supplementary Table 2). The GC content of the two chloroplast genomes was uneven, with approximately 29.5% for *C. palangshanense* and 35.4% for *C. debile*. The GC content of the two chloroplast genomes varied considerably in the LSC region (24.7% and 32.7%, respectively) while similar in the IRs regions (38.6% and 40.1%, respectively) and in the SSC regions (both 29.0%) (Table 1). Accordingly, *C. palangshanense* had high proportions of A (37.6%) and T (37.8%) nucleotides and low proportions of G (11.6%) and C (12.1%) nucleotides in the LSC region, while the base/nucleotide composition in the SSC and IRs regions were similar to other five species (Supplementary Figure 1 and Supplementary Table 2).

**TABLE 1:** General characteristics of the chloroplast genomes of the seven *Cypripedium* species.

Species	*C. palangshanense*	*C. debile*	*C. subtropicum*	*C. tibeticum*	*C. japonicum*	*C. formosanum*	*C. calceolus*
GenBank No.	MW924110	MW924111	MT937100*	MT937101*	NC_027227*	NC_026772*	NC_045400*
Total length (bp)	207,142	162,773	212,668	197,815	174,417	178,131	175,122
Length of LSC (bp)	128,862	89,446	129,998	117,193	97,322	100,973	97,486
Length of SSC (bp)	9,450	10,049	27,414	25,094	21,911	21,921	22,260
Length of IR (bp)	34,415	31,639	27,628	27,764	27,592	27,579	27,688
Total GC content (%)	29.5	35.4	28.2	30.5	34.5	33.9	34.4
LSC GC content (%)	24.7	32.7	23.7	26.5	31.7	30.7	31.6
IR GC content (%)	38.6	40.1	42.6	42.5	42.7	42.7	42.6
SSC GC content (%)	29.0	29.0	20.6	22.4	26.4	26.4	26.1
Number of genes	128 (20)	130(20)	131 (20)	132(20)	131(20)	132(20)	132(20)
Number of CDs genes	82(8)	84(8)	85 (8)	86 (8)	85 (8)	86 (8)	86 (8)
Number of tRNA genes	38(8)	38(8)	38 (8)	38 (8)	38 (8)	38 (8)	38 (8)
Number of rRNA genes	8 (4)	8 (4)	8 (4)	8 (4)	8 (4)	8 (4)	8 (4)

*Sequences downloaded from GenBank. Numbers in brackets indicate genes duplicated in the IR regions.

The chloroplast genomes of *Cypripedium palangshanense* and *C. debile* consisted of 128–130 genes, including 82–84 protein-coding genes, 38 tRNA genes, and 8 rRNA genes (Table 1). Each IR contain four rRNA genes (*rrn4.5*, *rrn5*, *rrn16*, *rrn23*), eight tRNA genes (*trnH*-*GHG*, *trnL*-*CAU*, *trnL*-*CAA*, *trnV*-*GAC*, *trnL*-*GAU*, *trnA*-*UGC*, *trnR*-*ACG*, *trnN*-*GUU*) and eight protein-coding genes (*rps7*, *rps12*, *rps19*, *rpl2*, *rpl23*, *ndhB*, *ycf1*, *ycf2*; Supplementary Table 3). A total of eleven genes contained one intron, including eight protein-coding genes (*rps16*, *rpl2*, *rpl16*, *rpoC1*, *petB*, *petD*, *ndhA*, *ndhB*) and three tRNA genes (*trnG*-*UCC*, *trnK*-*UUU*, *trnA*-*UGC*). Three protein-coding genes (*atpF*, *rps12*, *clpP1*) and three tRNA genes (*trnL*-*UAA*, *trnV*-*UAC*, *trnA*-*UGC*) contained two introns, while *ycf3* gene which has four introns (Supplementary Table 3).

### Interspecies Plastids Sequence Analysis and Highly Variable Regions Identification

**FIGURE 2:**
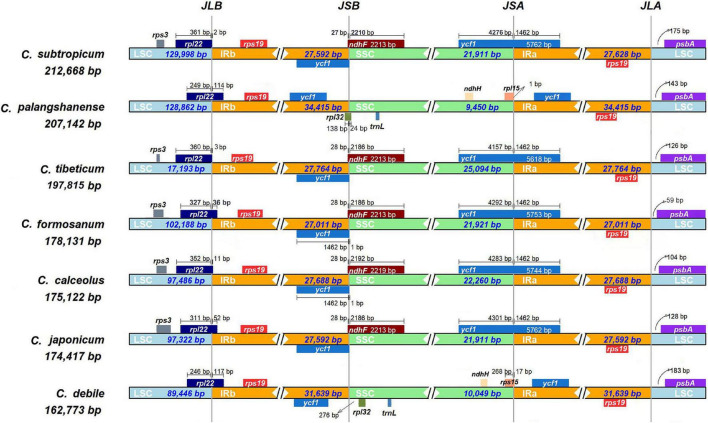
Analyses of expansion and contraction of inverted repeats in the seven *Cypripedium* chloroplast genomes.

The chloroplast genomes of seven *Cypripedium* species showed a significant difference in genome size (162,773–212,668 bp; Table 1 and Supplementary Figure 2) and a 75-kb inversion (*trn*G-UCC to *trn*P-UGG) occurred in the LSC region for three species: *C. subtropicum*, *C. tibeticum* and *C. formosanum* (Supplementary Figure 2). The whole aligned sequences show high variability in the LSC and SSC regions, and high similarities in IRs except the *ycf*2-*ndh*B regions’ sequence identities significantly falling below 50% (Supplementary Figure 3). The LSC/IRb (JLB: *rpl22*) boundary and the LSC/IRa (JLA: *rps19* & *psbA*) boundary were stable among the seven *Cypripedium* chloroplast genomes. However, the two newly sequenced species show great differences in the locations of the SSC/IRb (JSB) boundary and the SSC/IRa (JSA) boundary compared to previously published species. The JSB boundary was located between *ycf1* gene and *rpl32* gene in *C. debile* and exclusively on the *rpl32* gene in *C. palangshanense*, while stable (*ycf1* & *ndhF*) in other five species. The JSA boundary was located on the *rpl15* gene in both *C. debile* and *C. palangshanense*, while on the *ycf1* gene in the other five species (Figure 2).

**FIGURE 3:**
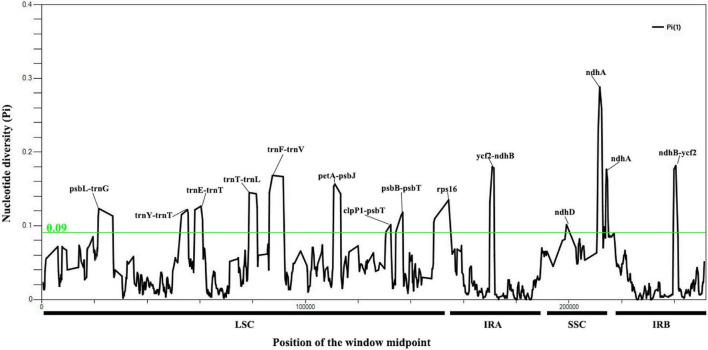
Nucleotide variability values compared between the seven chloroplast genomes of *Cypripedium* using the window sliding analysis. (*X*-axis indicates the position of the midpoint of the window, while *Y*-axis indicates the nucleotide diversity of each window).

A total of 7,929 variable (polymorphic) sites were found in 251,932 nucleotide loci, including 5,411 singleton variable sites (SVS) and 2,518 parsimony informative sites (PIS). Three different categories under SVS were observed, 5,133 sites with two variants (SV2V), 275 sites with three variants (SV3V) and 3 sites with four variants (SV4V). Similarly, PIS has three categories: 2,265 sites with two variants (PIS2V), 240 sites with three variants (PIS3V) and 13 sites with four variants (PIS4V; Supplementary Table 4). The window-based nucleotide variability (Pi) values for the alignment of the seven chloroplast genomes ranged from 0 to 0.28833. We identified 12 highly divergent regions (Pi > 0.09) with Pi values ranging from 0.09 to 0.28833, including nine intergenic spacer (IGS) regions (*psbL*-*trnG*, *trnY*-*trnT*, *trnE*-*trnT*, *petA*-*psbJ*, *clpP1*-*psbT*, *psbB*-*psbT*, *ycf2*-*ndhB*, *trnT*-*trnL*, and *trnF*-*trnV*) and three coding sequence regions (*ndhD*, *ndhA* and *rps16*; Figure 3; Supplementary Table 5).

### Repeat Sequences Analysis

**FIGURE 4:**
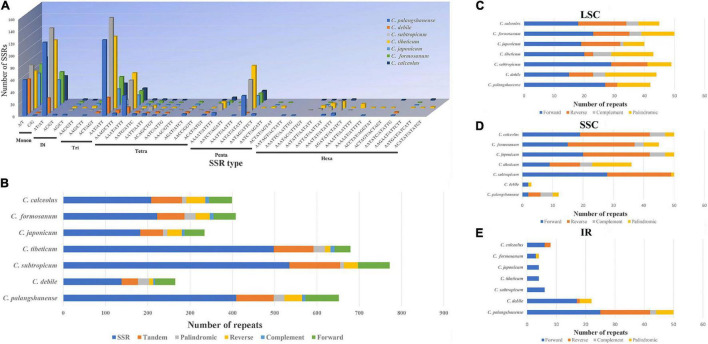
Analyses of repeat sequences in seven chloroplast genomes. **(A)** Number of different SSRs types. **(B)** Number of SSRs, tandem repeats and large LSRs. **(C)** Number of different LSRs types in the LSC regions. **(D)** Number of different LSRs types in the SSC region. **(E)** Number of different LSRs types in the IR region.

A total of 2,192 SSRs were identified in the seven *Cypripedium* chloroplast genomes (Figure 4A and Supplementary Table 6). The mononucleotide, dinucleotide, trinucleotide, tetranucleotide, pentanucleotide, and hexanucleotide of these SSRs were account for 20.2, 24.7, 25.0, 13.5, 12.4 and 4.2%, respectively. The five dominant motif types in the SSRs were A/T, AT/TA, AAT/ATT, AAAT/ATTT, and AATAT/ATATT. Among 41 different SSR types, *C. palangshanense* had 3 unique types, *C. subtropicum* and *C. tibeticum* had 2 unique types, while *C. formosanum* had one unique type (Figure 4A and Supplementary Table 6). A total of 786 large sequence repeats (LSRs; ≥ 30 bp and Hamming distance = 3) were identified in the seven *Cypripedium* chloroplast genomes (Figure 4B and Supplementary Table 7). In general, F repeats (386) were the most common type, while C repeats (54) were the least. Among the seven species, *C. japonicum* (87) contained the least LSRs, and *C. palangshanense* contained the most (154; Figure 4B and Supplementary Table 7). In the LSC region, the seven *Cypripedium* species had similar numbers of LSRs (40–50) but showed significant differences in the SSC (3–50) and IR (4–50) regions (Figures 4C-E and Supplementary Table 8).

### Phylogenetic Analysis

**FIGURE 5:**
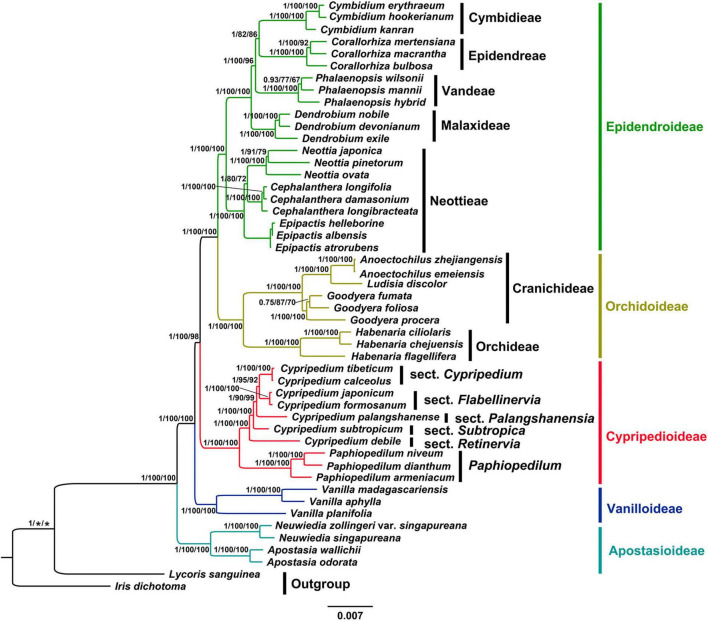
Phylogram depicting the relationships of the plastid CDSs of 47 different Orchidaceae species using ML method. Numbers associated with nodes indicated Bayesian posterior probabilities (PP), maximum likelihood (BS_ML_) and maximum parsimony (BS_MP_) bootstrap values, respectively. The PP on the left, BS_ML_ in the middle, BS_MP_ on the right.

The phylogenetic analysis using MP, BI and ML methods based on whole chloroplast genomes (IRa excluded) and CDSs yielded identical topologies with high support values (Figure 5 and Supplementary Figures 4–8). Thus, we present the topology resulting from ML analysis based on CDSs, with posterior probability (PP), maximum likelihood (BS_ML_) and maximum parsimony (BS_MP_) bootstrap values labeled on the tree branches. Our phylogenetic tree indicated that the subfamily Apostasioideae (PP = 1, BS_ML_ = 100%, BS_MP_ = 100%) diverged first and was sister to remaining taxa of the orchid family, followed by Vanilloideae (PP = 1, BS_ML_ = 100%, BS_MP_ = 100%), which is sister to a strongly supported group (PP = 1, BS_ML_ = 100%, BS_MP_ = 98%) comprising Cypripedioideae (PP = 1, BS_ML_ = 100%, BS_MP_ = 100%), Orchidoideae (PP = 1, BS_ML_ = 100%, BS_MP_ = 100%) and Epidendroideae (PP = 1, BS_ML_ = 100%, BS_MP_ = 100%). Within the subfamily Cypripedioidea, we resolved a strongly supported clade consisting of the genus *Paphiopedium* (PP = 1, BS_ML_ = 100%, BS_MP_ = 100%) and *Cypripedium* (PP = 1, BS_ML_ = 100%, BS_MP_ = 100%). Our phylogenetic analyses also strongly supported (PP = 1, BS_ML_ ≥ 90%, BS_MP_ ≥ 92%) the monophyly of the sections of genus *Cypripedium*, sect *Cypripedium*, *Flabellinervia*, *Subtropica*, *Retinervia* and *Palangshanensia*.

## Discussion

### Characterization of Seven Chloroplast Genomes in *Cypripedium*

The chloroplast genome of the newly generated *Cypripedium debile* in this study is the smallest in the genus, while the previously published genome of *C. subtropicum* remains the largest in the genus, as well as in the Orchid family. The chloroplast genomes of *Cypripedium* (162,773–212,668 bp) were larger than the average of sequenced land plants (151 kb), while the GC content (28.2–35.4%) was lower than the average of sequenced land plants (37.6%; Guo et al., 2021). The IR regions of the two newly reported chloroplast genomes (31,639–34,415 bp) were significantly larger than five published *Cypripedium* (27,579–27,764 bp), while the SSC regions were the opposite (9,450–10,049 bp and 27,414–31,911 bp; Table 1). This indicates the expansion of IR into SSC region has contributed the most to the decrease of SSC region in *C. debile* and *C. palangshanense*, which has been reported in *Corydalis* species (Xu and Wang, 2021).

Among the seven species, three species (*Cypripedium tibeticum*, *C. palangshanense* and *C. subtropicum*, 197,815–212,668 bp) with larger chloroplast genomes had lower GC content (28.2–30.5%), while the other four species (*C. debile*, *C. japonicum*, *C. calceolus* and *C. formosanum*, 162,773–178,131 bp) with smaller chloroplast genomes had higher GC content (33.9–35.4%). In addition, we find all *Cypripedium* species had much lower GC content in the LSC and SSC regions (23.7–31.7% and 20.6–29.0%, respectively) than in the IR region (38.6–42.7%) due to the reduction of AT nucleotides in the four rRNA genes (*rrn23*s, *rrn16*s, *rrn4.5*s, and *rrn5*s), which is also reported in Meng et al. (2018); Hishamuddin et al. (2020). Meanwhile, this study suggested that the chloroplast genomes of *Cypripedium* showed very high level of A + T content (64.6%–71.8%) and low level of G + C content (28.2%–35.4%), a feature rarely observed in chloroplast genome sequences of many land plants (Bi et al., 2018). The high AT content was caused by repetitive sequences consisting of poly (A), poly (T) or poly (AT) regions in the non-coding regions of the single-copy regions, especially in the LSC region (Kim et al., 2015).

The gene order of the two newly sequenced chloroplast genomes in this study is conserved without gene rearrangement and inversion events, but a long inversion (75-kb) occurred in the LSC region in three published *Cypripedium* species (*C. subtropicum*, *C. tibeticum* and *C. formosanum*), resulted in very high variability of this region in the whole aligned sequences (Supplementary Figures 2, 3). Interestingly, remarkable divergence was also observed in the SSC region, where no inversion event occurred (Supplementary Figure 3). Meanwhile, we found that four *ndh* genes (*ndhE*, *ndhF*, *ndhH*, and *ndhI*) in *C. palangshanense* disappeared in the SSC region compared to *C. tibeticum*, *C. japonicum*, *C. calceolus*, *C. formosanum*, and *C. subtropicum*, as well as three *ndh* genes (*ndhF*, *ndhG* and *ndhH*) in *C. debile* were lost in the SSC region (Figure 1). A study by Lin et al. (2015) showed that in *C. formosanum*, the *ndh* genes transferred from the chloroplast genome to the mitochondrial genome. Thus, a similar phenomenon may occur in the two newly sequenced species. Also, different degrees of loss or deletion of the *ndh* genes among species in *Cypripedium* resulted in significant variability in the SSC region. Furthermore, gene loss or pseudogenization has partially counteracted the increase of chloroplast genomes size in this genus, which showed a similar expansion-contraction mechanism in *Corydalis* (Xu and Wang, 2021) and *Pelargonium* (Weng et al., 2017).

### Identification of Polymorphic Loci for Molecular Markers

Highly variable regions have potential for species identification and wide-range phylogenetic analysis (Zhang et al., 2009). In the present study, the *ndhA* located in the SSC region had the highest nucleotide variation (Pi = 0.28833; Figure 3), which may be associated with the expansion and contraction of IRs (Goulding et al., 1996) and the transfer of *ndh* from the chloroplast genome to the mitochondrial genome (Lin et al., 2015). The IR region is relatively conserved except in the region between *ycf2* and *ndhB* (Figure 3 and Supplementary Figure 3). The introns of *rps16* and intergenic regions with relatively high divergence values located in the LSC region (*psbL*-*trnG*, *trnY*-*trnT*, *trnE*-*trnT*, *trnT*-*rnL*, *trnF*-*trnV*, *petA*-*psbJ*, *clpP1*-*psbT*, and *psbB*-*psbT*) were associated with repetitive sequences (Figure 3). For example, a various number of repetitive sequences can be found in the IGS region of *psbL*-*trnG*, *trnF*-*trnV*, *trnT*-*trnL* and *petA*-*psbJ*. The highly polymorphic regions identified in this study have potential to be exploited as candidate barcode sequences in the phylogenetic analysis of *Cypripedium*. Further work is needed to verify whether these markers can be recommended as valid barcodes for species of the genus *Cypripedium*.

### Repeat Sequences Analysis

SSRs (≥ 10 bp) are small repetitive units of chloroplast DNA, together with LSRs, have played an important role in the evolution of the chloroplast genome and may contribute to the development of future molecular markers (Zhang et al., 2016; Li et al., 2021). Interestingly, the three larger chloroplast genomes (*Cympripedium tibeticum*, *C. palangshanense*, and *C. subtropicum*; 98–154 LSRs, and 409–535 SSRs; Figure 4B and Supplementary Table 7) had more LSRs and SSRs than the four smaller ones (*C. debile*, *C. japonicum*, *C. calceolus*, and *C. formicum*; 87–114 LSRs, and 138–222 SSRs). This indicates increase of LSRs and SSRs led to the obvious enlargement of these chloroplast genomes (*C. tibeticum*, *C. palangshanense*, and *C. subtropicum*). Moreover, in the IR region, the number of LSRs of the two newly sequenced chloroplast genomes (22–50) is approximately six times higher than the five published plastids (4–8). At the SSC region, the number of LSRs of the two newly sequenced chloroplast genomes (3–12) are approximately one-fifth of the five published plastids (36–50). Thus, we speculate that the increase of LSRs in the IR regions of *C. debile* and *C. palangshanense* led to the marked enlargement of their IR regions, while the decrease of LSRs in the SSC region of *C. debile* and *C. palangshanense* that led to the significantly smaller of their SSC region. Furthermore, we find that the coding region of this genus is conserved and that chloroplast genome expansion is closely associated with the proliferation of IGS regions, especially in the LSC region, which is also reported in Guo et al. (2021). And frequent variation in the repeat region also plays an important role in the variation and sequence rearrangement of the chloroplast genome (Zhang et al., 2016; Yuan et al., 2017).

### Phylogenetic Analysis

Although chloroplast genome data-set has been considered as a single locus due to their uniparental inheritance, a growing number of studies indicate the implementation of complete chloroplast genome data-set has the potential to resolve the phylogenetic relationships of controversial genus (Wolfe et al., 1987; Green, 2011; Wicke et al., 2011; Schwarz et al., 2017; Gonçalves et al., 2019; Zhang et al., 2020). Our phylogenetic tree based on both whole chloroplast genomes (IRa excluded) and CDSs sequences resolved *Cypripedium* as a monophyletic taxon with high support values (PP = 1, BS_ML_ = 100%, BS_MP_ = 100%), which is consistent with the results from the plastid markers (*matK, rbcL*, *trnH-psbA*, *trnS-trnfM*, *atpI-atpH*, vspace*-0.5pttextittrnL intron, and *trnL-F*; Cox et al., 1997; Fatihah et al., 2011; Li et al., 2011) and nuclear ribosomal ITS analyses (Fatihah et al., 2011; Szlachetko et al., 2020). The inter-section relationships of the genus were resolved as: (sect. *Retinervia* (*Subtropica* (*Palangshanensia* (*Flabellinervia*, *Cypripedium*)))). However, the systematic position of sect. *Retinervia* and *Palangshanensia* has been controversial. The traditional *Retinervia* (*C. debile*, *C. palangshanense* and *C. elegans* Reichenbach) is described by Cribb (1997) based on the following morphological characteristics: two opposite leaves, situated near the middle of the stem and produce a single-flowered inflorescence terminated by the smallest flower within the genus with tepals distinctly longer than the lip. Recently, Li et al. (2011) firstly showed that *C. palangshanense* is not included in the sect. *Retinervia* based on five plastid markers and nuclear gene. Then Chen et al. (2013) first proposed to separate *C. palangshanense* from the sect. *Retinervia* to create sect. *Palangshanensia* based on the phylogenetic results of Li et al. (2011). Finally, both results from ITS/ACO (BS = 84, PP = 1.0; Szlachetko et al., 2020) and our whole chloroplast genomes (IRa excluded) and CDSs analysis support the establishment of the sect. *Palangshanensia*. Unfortunately, *C. elegans* of this section was not included in this study, and further studies are needed to verify its systematic position in the future.

The results of the phylogenetic inference based on whole chloroplast genomes (IRa excluded) and CDSs support the relationships between the five subfamilies of the orchid family: Apostasioideae, Vanilloideae, Cypripedioideae, Orchidoideae, and Epidendroideae, which is consistent with previous studies (Cameron et al., 1999; Guo et al., 2012; Chase et al., 2015; Deng et al., 2015; Givnish et al., 2015; Li et al., 2016, 2019). In the present study, the subfamily Apostasioideae (including *Neuwiedia* and *Apostasia*) diverged firstly and is sister to all the other subfamilies in Orchidaceae, which is congruent with morphological characters (Pridgeon et al., 1999). Subfamily Vanilloideae diverged secondly, followed by subfamily Cypripedioideae, which is consistent with previous studies (Guo et al., 2012; Chase et al., 2015; Deng et al., 2015; Givnish et al., 2015; Li et al., 2016). The subfamily Orchidoideae, consisting of approximately 190 genera and 3600 species, is the second largest subfamily within Orchidaceae (Li et al., 2016). The researchers reached a consensus on the four orchid genera sampled in this study (Cameron et al., 1999; Li et al., 2016), which were divided into two clades, Cranichideae and Orchidaceae. The subfamily Epidendroideae is the most complicated taxon, representing a greater number of genera (ca. 650 genera) and species (ca. 18,000 species) than the total number of the other four subfamilies (Li et al., 2016). In this study, we selected several genera of key nodes in the subfamily and revealed their phylogenetic relationships. Our phylogenetic tree indicated that the tribe Neottieae diverged as sister to the remaining Epidendroideae, followed by the tribe Malaxideae, which is sister to three well-supported tribes comprising Vandeae, Epidendreae and Cymbidieae. However, the relationship among these three tribes has been controversial as well. Chase et al. (2015); Li et al. (2016) supported the topology (Cymbidieae (Vandeae, Epidendreae)) based on low-copy nuclear gene (*Xdh*) and plastid markers (*rbcL*, *matK*, *psaB*, *ycf1*). Givnish et al. (2015); Li et al. (2019) supported another topology (Epidendreae (Vandeae, Cymbidieae)) based on plastid and mitochondrial genomes. Thus, future studies with extensive taxon sampling and molecular and/or morphological evidence are needed to provide a higher resolution of the relationships among three tribes in this subfamily.

## Conclusion

In the present study, we reported the complete chloroplast genomes of two newly sequenced *Cypripedium* species for comparative genomic analysis with five other published species. We revealed the mechanism of significant genome amplification of this genus and discussed that expansion of the IR region leads to gene pseudogenization or loss in the SSC region. Also, identification of polymorphic loci and molecular markers was performed, which will be useful for species identification and determination of phylogenetic relationships in the future. We made efforts to enrich the genomic resources of *Cypripedium*, which may help to promote the conservation of these endangered species. Meanwhile, the data-sets of the whole chloroplast genomes (IRa excluded) and CDSs sequences provided new insights in addressing the phylogeny of Orchidaceae, as well as genetic resources for further phylogenetic studies for this family. Future studies should be complemented by larger sample sizes to elucidate the phylogenetic relationship of these species.

## Data Availability Statement

The chloroplast genome sequences of *Cypripedium palangshanense* and *C. debile* were submitted to the National Center for Biotechnology Information (NCBI) and the accession numbers were: MW924110 and MW924111, respectively. Raw reads were submitted to the NCBI database under the BioProject number: PRJNA838021.

## Author Contributions

BX and JZ designed the study. YC, WJ, and HD collected the plant materials. JZ, YF, AP-M, and XL performed the data analysis. JZ and ML drafted the manuscript. BX, ML, YF, and AP-M revised the manuscript. All authors reviewed and approved the final manuscript.

## Conflict of Interest

The authors declare that the research was conducted in the absence of any commercial or financial relationships that could be construed as a potential conflict of interest.

## Publisher’s Note

All claims expressed in this article are solely those of the authors and do not necessarily represent those of their affiliated organizations, or those of the publisher, the editors and the reviewers. Any product that may be evaluated in this article, or claim that may be made by its manufacturer, is not guaranteed or endorsed by the publisher.

## Footnotes

1
https://www.ncbi.nlm.nih.gov/genome/organelle/

2
http://www.dnabaser.com/

3
http://tree.bio.ed.ac.uk/software/figtree
