# Supplementary material for: Comparative Chloroplast Genomics of Seven Endangered Cypripedium Species and Phylogenetic Relationships of Orchidaceae
Source: Front Plant Sci. 2022 Jun 22;13:911702. doi: 10.3389/fpls.2022.911702 (PMC9257239; doi:10.3389/fpls.2022.911702)
Supplement: Supplementary file 1 [file Data_Sheet_1.docx]

Supplementary Material

# Supplementary Table

**Supplementary Table S1** List of used chloroplast genome sequences obtained from GenBank.

**Supplementary Table S2** The percentage (%) of each nucleotide (A, T, G, C) content in different regions.

**Supplementary Table S3** Annotated gene list of chloroplast genomes of seven *Cypripedium* species.

**Supplementary Table S4** Number of nucleotides in variable site and nucleotide diversity analysis among seven *Cypripedium* species.

**Supplementary Table S5** Thirteen regions of highly variable sequences in seven *Cypripedium*.

**Supplementary Table S6** Number of different SSRs types.

**Supplementary Table S7** Number and type of repeats.

**Supplementary Table S8** Number of large sequences repeats in different regions.

# Supplementary Figure

**Supplementary Figure 1** Base composition in the seven *Cypripedium* chloroplast genomes. The percentage (%) of each nucleotide (A, T, G, C) content in the large single-copy (LSC), small single-copy (SSC) and inverted repeats (IR) region, are shown.

**Supplementary** **Figure 2** Chloroplast genome alignment of 7 species of *Cypripedium*. The rectangles represent the locations of genes in each genome. White represents CDS, green represents tRNA, and red represents rRNA. The red boxes show the 75-kb inversion.

**Supplementary Figure 3** Visualization of alignment of seven *Cypripedium* species chloroplast genome sequences. VISTA-based identity plots showed sequence identity of seven chloroplast genomes with *Cypripedium subtropicum* (MT937100) as a reference. Genome regions are color coded as protein coding, rRNA coding (UTR), tRNA coding (UTR) or conserved noncoding sequences (CNS). The vertical scale indicates the percentage identity, ranging from 50 to 100%.

**Supplementary** **Figure 4** Phylogenetic tree obtained using the Bayesian inference (BI) method of the plastid CDSs of 47 different Orchidaceae species. Numbers above branches indicate Bayesian posterior probabilities (PP).

**Supplementary** **Figure 5** Phylogenetic tree obtained using the Maximum parsimony (MP) method of the plastid CDS of 47 different Orchidaceae species. Numbers above branches indicate MP bootstrap supports (BS_MP_).

**Supplementary** **Figure 6** Phylogenetic tree obtained using the Maximum likelihood (ML) method of the plastid genomes of 47 different Orchidaceae species. Numbers above branches indicate ML bootstrap supports (BS_ML_).

**Supplementary** **Figure 7** Phylogenetic tree obtained using the Bayesian inference (BI) method of the plastid genomes of 47 different Orchidaceae species. Numbers above branches indicate Bayesian posterior probabilities (PP).

**Supplementary** **Figure 8** Phylogenetic tree obtained using the Maximum parsimony (MP) method of the plastid genomes of 47 different Orchidaceae species. Numbers above branches indicate MP bootstrap supports (BS_MP_).
