# Supplementary figures and images for: Comparative Chloroplast Genomics of Seven Endangered Cypripedium Species and Phylogenetic Relationships of Orchidaceae
Source: Front Plant Sci. 2022 Jun 22;13:911702. doi: 10.3389/fpls.2022.911702 (PMC9257239; doi:10.3389/fpls.2022.911702)

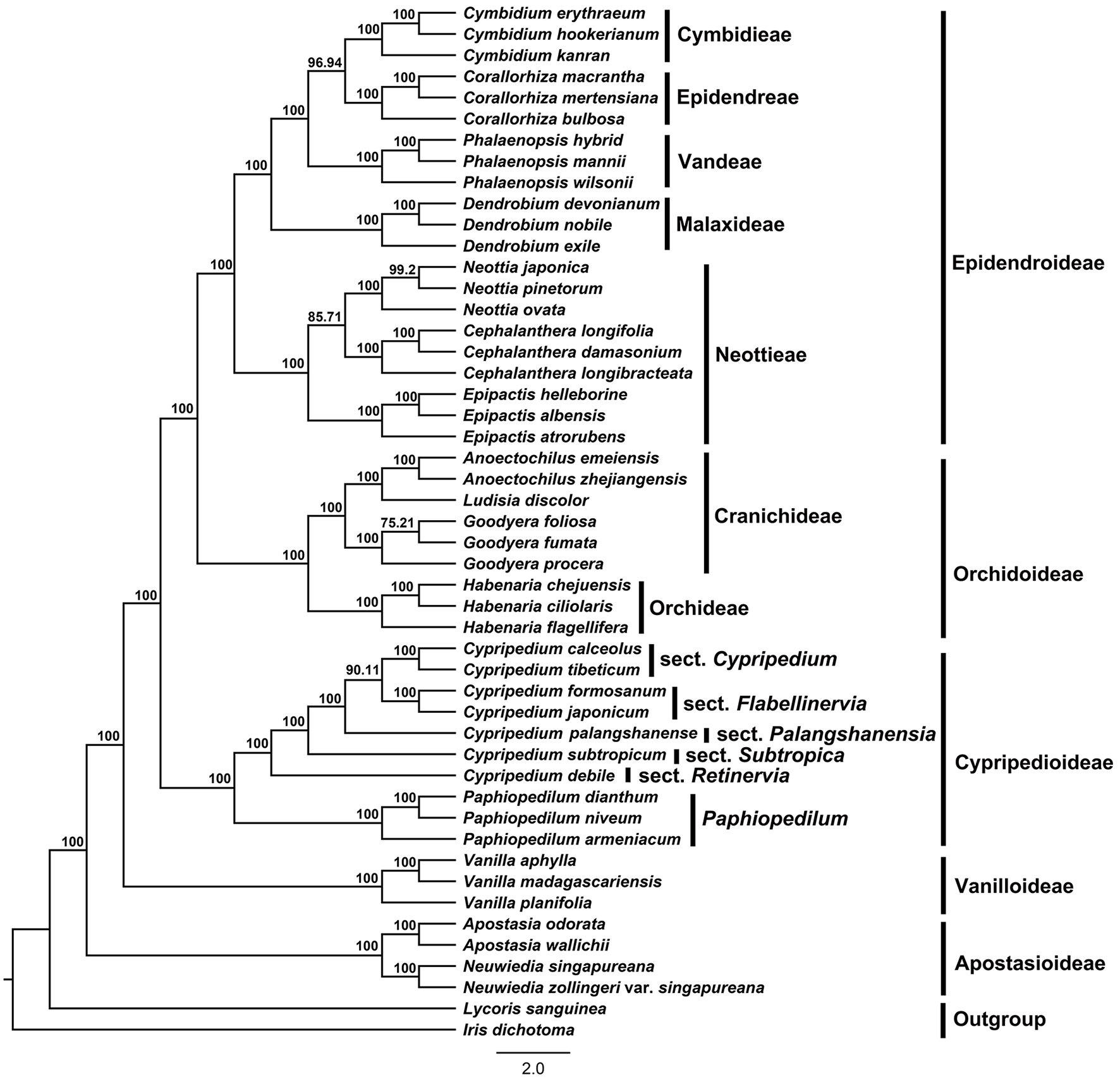

Supplement: Supplementary file 4 [file Image_1.TIF]

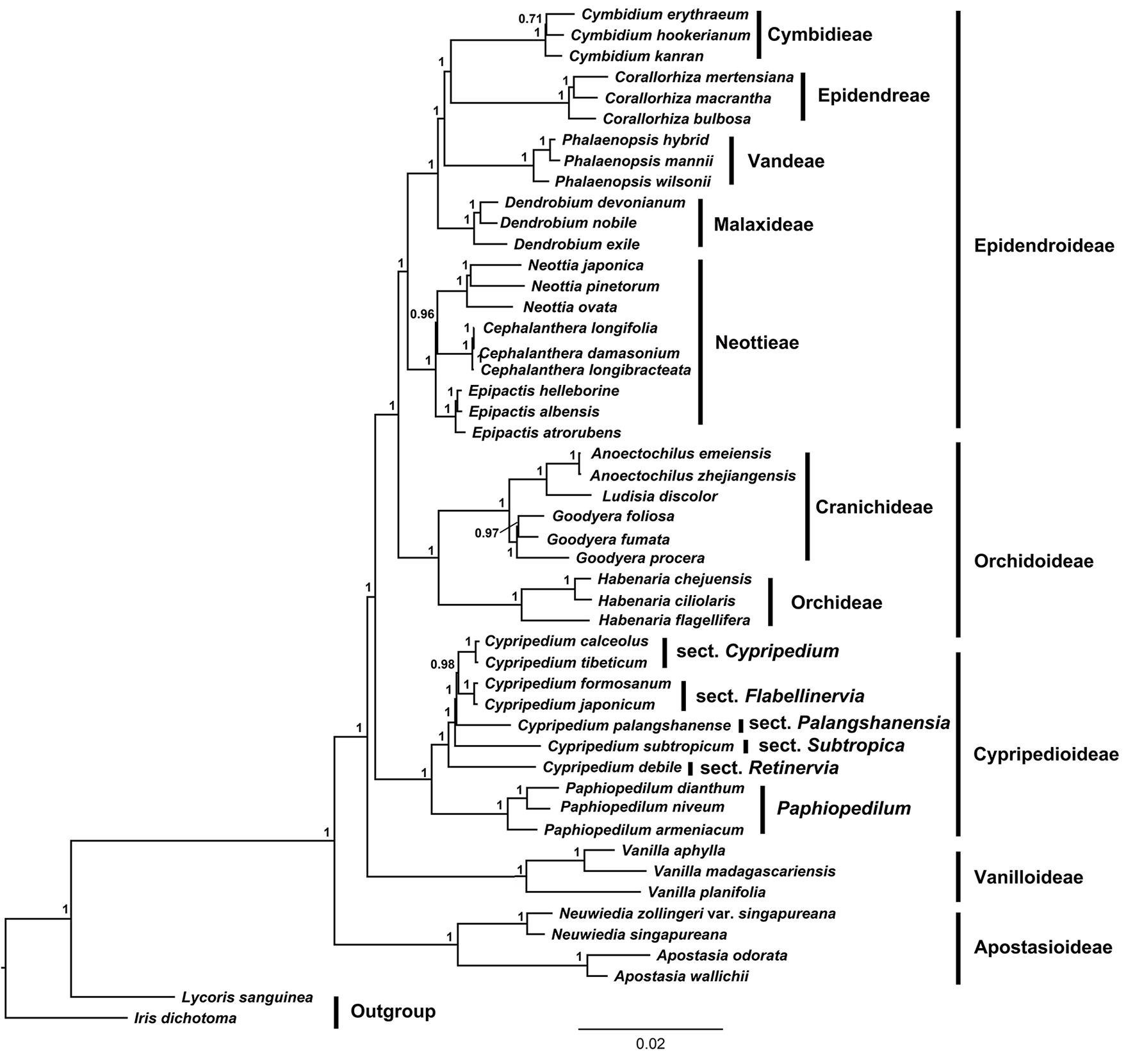

Supplement: Supplementary file 5 [file Image_2.TIF]

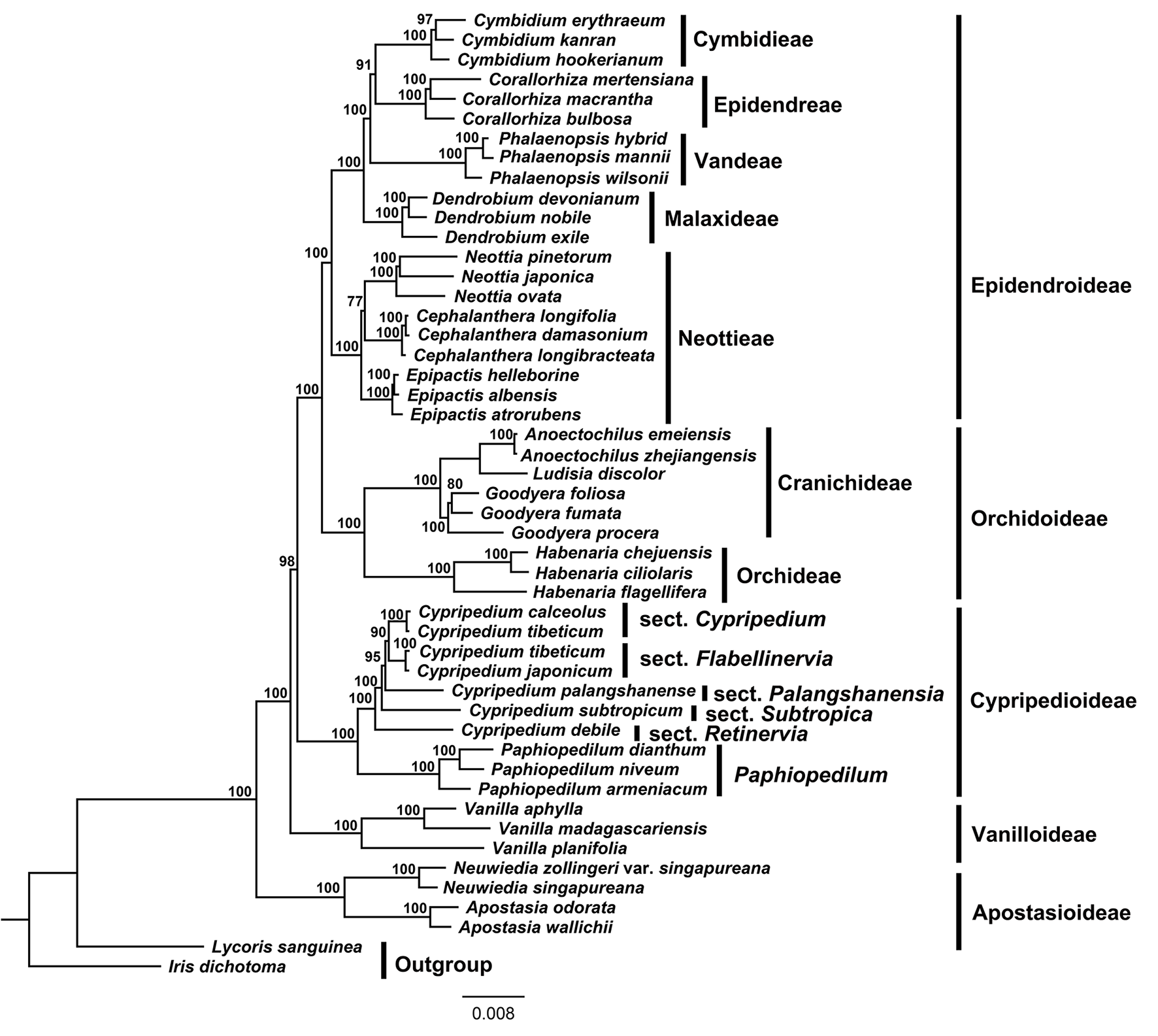

Supplement: Supplementary file 6 [file Image_3.TIF]

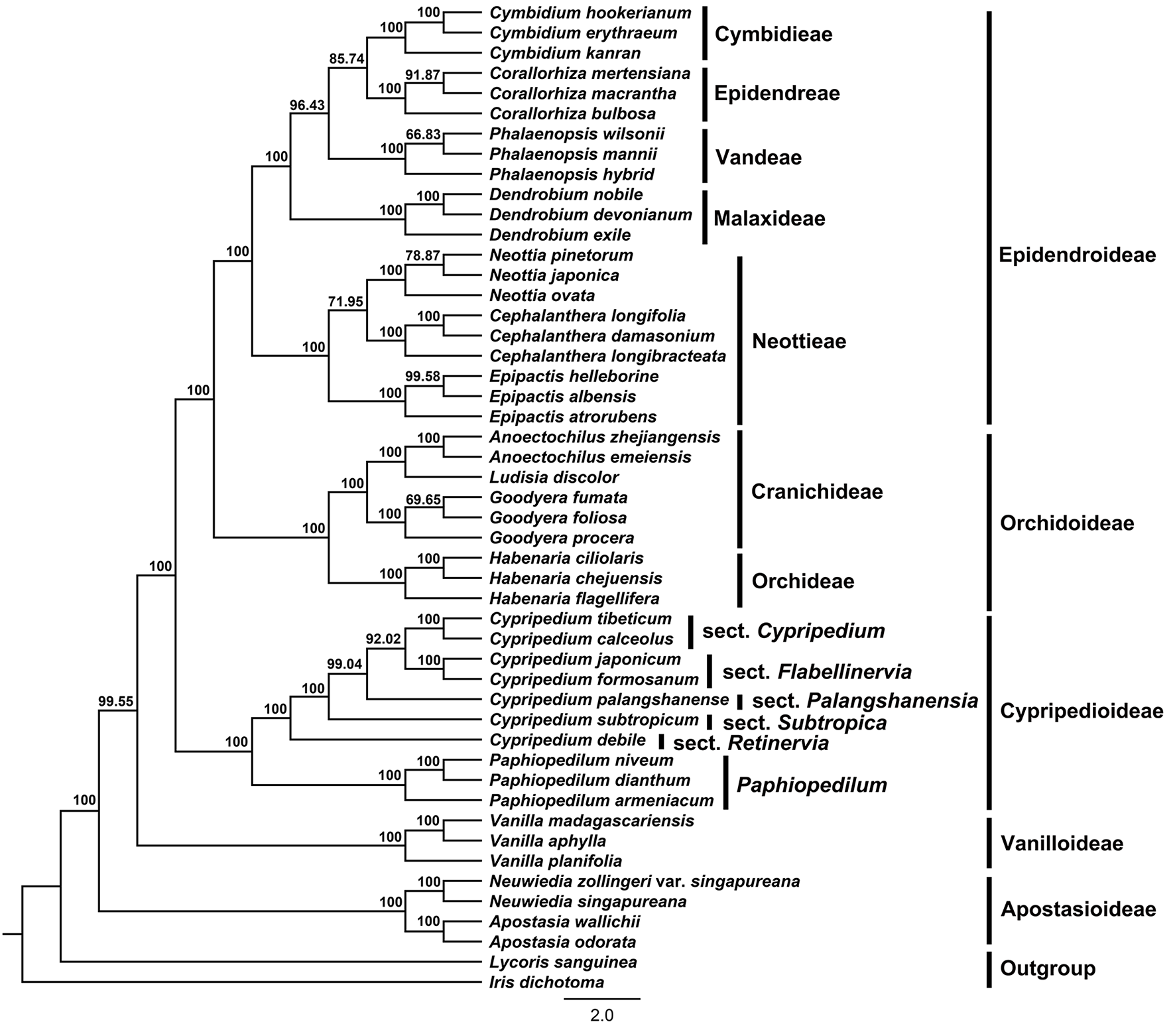

Supplement: Supplementary file 7 [file Image_4.TIF]

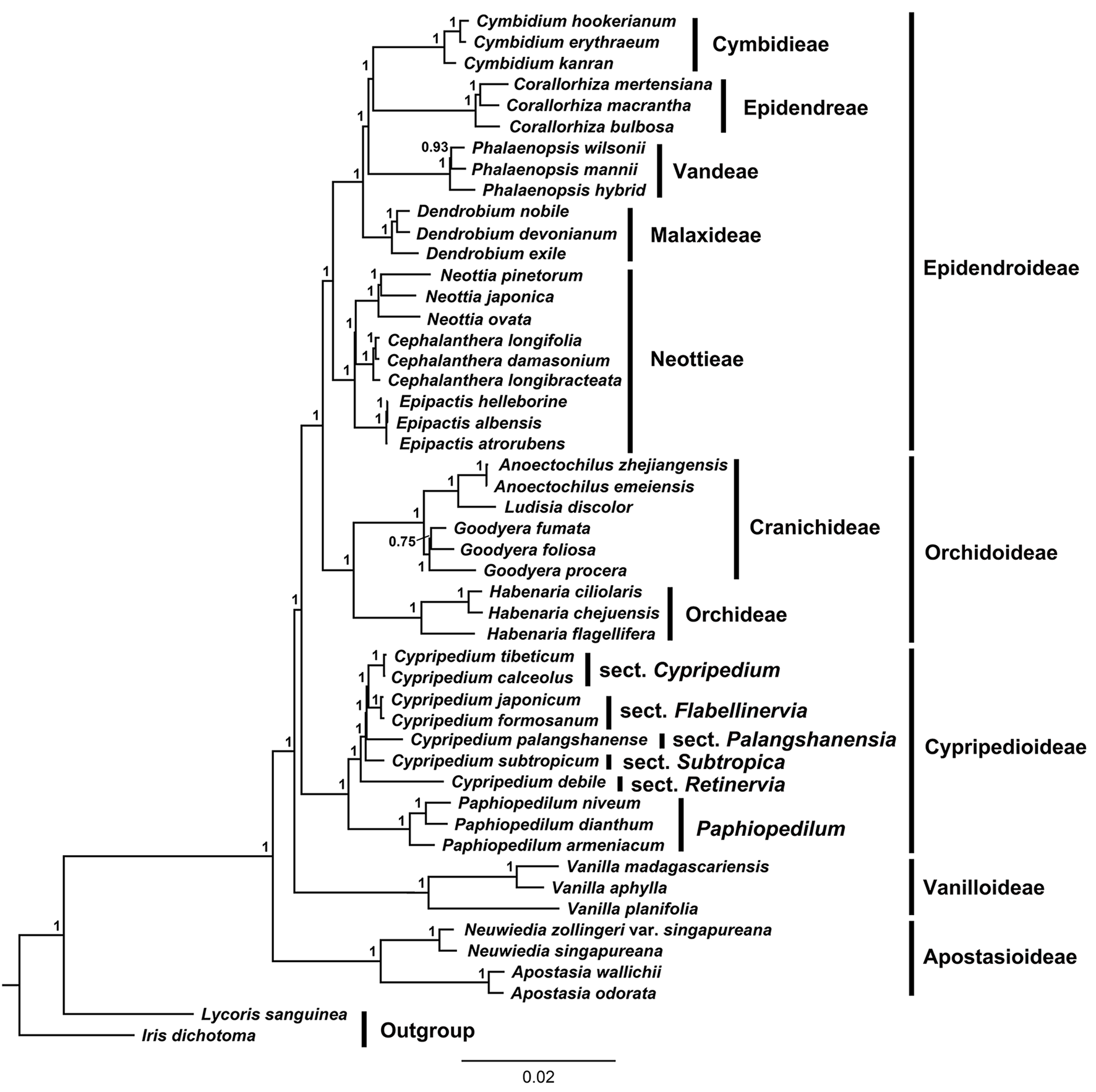

Supplement: Supplementary file 8 [file Image_5.TIF]

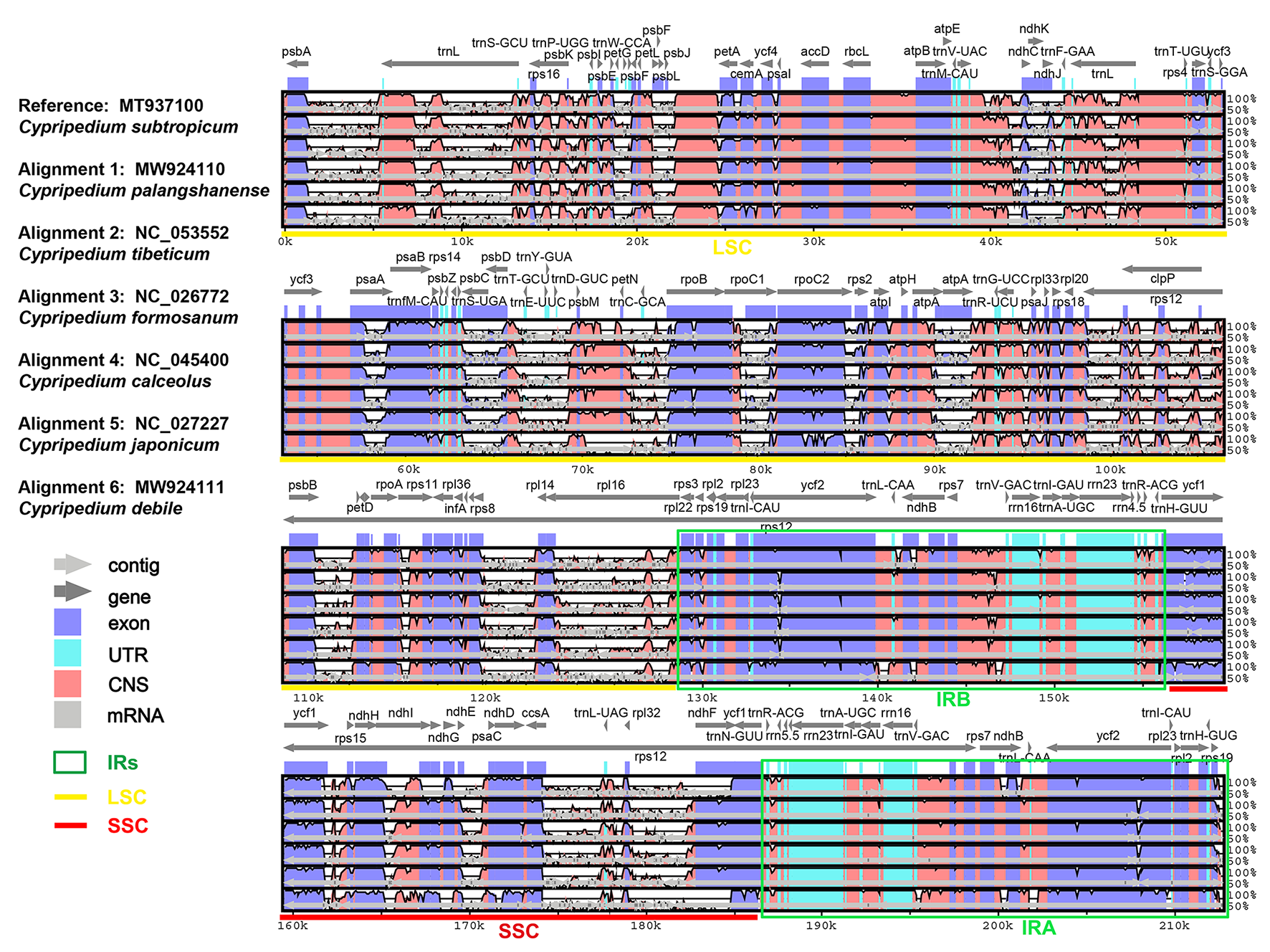

Supplement: Supplementary file 9 [file Image_6.TIF]

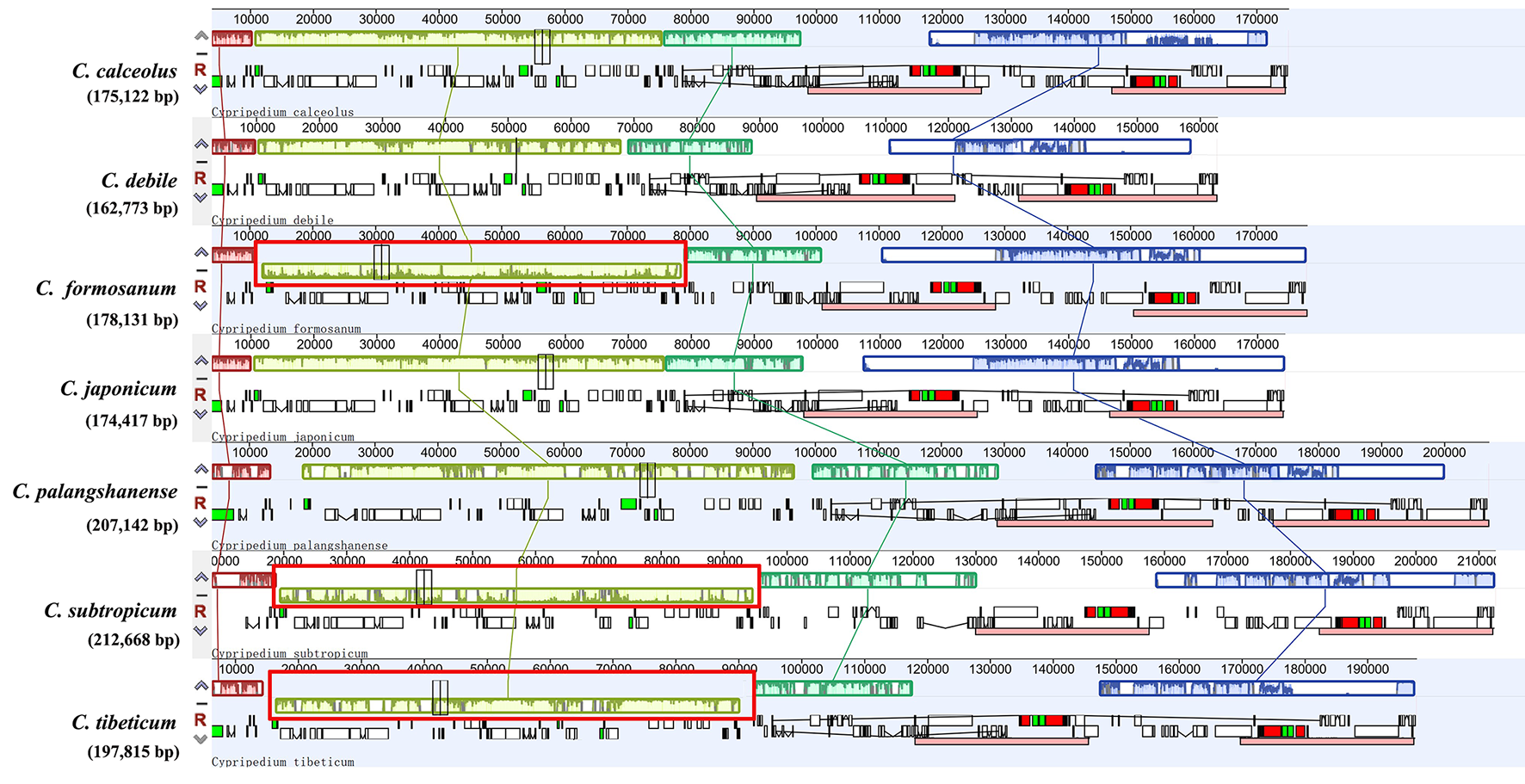

Supplement: Supplementary file 10 [file Image_7.TIF]

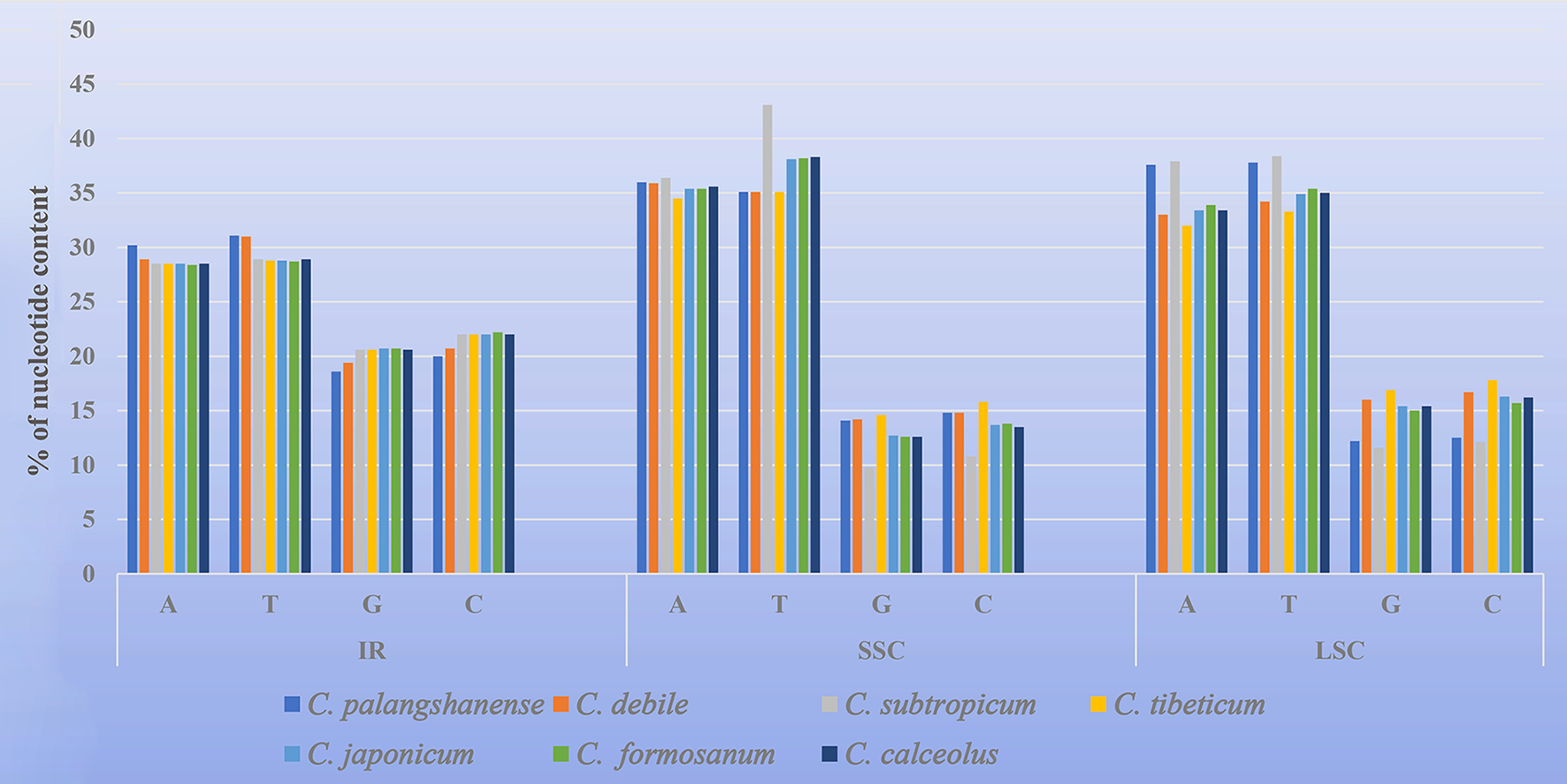

Supplement: Supplementary file 11 [file Image_8.TIF]
